# Comparison of the impact of orthodontic treatment and orthognathic surgery on oral health-related quality of life in Iranian female patients

**DOI:** 10.21142/2523-2754-1404-2026-312

**Published:** 2026-10-01

**Authors:** Fahimeh Farzanegan, Hooman Shafaee, Zahra Hosseini, Peyman Zamanipour

**Affiliations:** 1 Department of Orthodontics, School of Dentistry, Mashhad University of Medical Sciences, Mashhad, Iran. farzaneganf@mums.ac.ir shafaeeH@mums.ac.ir zmppeyman@gmail.com Mashhad University of Medical Sciences Department of Orthodontics School of Dentistry Mashhad University of Medical Sciences Mashhad Iran farzaneganf@mums.ac.ir shafaeeH@mums.ac.ir zmppeyman@gmail.com; 2 General dentist, Private practice, Mashhad, Iran. HosseiniZ903@mums.ac.ir General dentist Private practice Mashhad Iran HosseiniZ903@mums.ac.ir

**Keywords:** oral health-related quality of life, orthodontic treatment, orthognathic surgery, child perception questionnaire, orthognathic quality of life questionnaire, dentofacial deformity, calidad de vida relacionada con la salud bucal, tratamiento de ortodoncia, cirugía ortognática, cuestionario de percepción infantil, cuestionario de calidad de vida ortognática, deformidad dentofacial

## Abstract

**Objective:**

Oral health-related quality of life (OHRQoL) is a critical outcome in treating dentofacial discrepancies. This prospective cohort study aimed to compare OHRQoL improvements three months after completion of fixed orthodontic treatment versus orthognathic surgery among Iranian female patients.

**Materials and Methods:**

A total of 101 female patients were included: 71 adolescents (aged 14-17 years) undergoing fixed orthodontic treatment and 30 young adults (aged 19-35 years) undergoing combined orthodontic-orthognathic surgery for skeletal malocclusion. OHRQoL was assessed at baseline and three months post-treatment using the Persian versions of the Child Perception Questionnaire (CPQ) for both groups and the Orthognathic Quality of Life Questionnaire (OQLQ) for the surgical group. Within- and between-group changes were analyzed using paired and independent t-tests (p < 0.05).

**Results:**

Both treatments significantly improved overall OHRQoL. The orthodontic group showed significant reductions in all four CPQ domains and total score (p < 0.001). The surgical group exhibited significant improvements in emotional well-being, social well-being, and total CPQ score (p < 0.001), but not in oral symptoms (p = 0.213) or functional limitations (p = 0.284). OQLQ scores in the surgical group improved across all domains (p < 0.01).

**Conclusion:**

At three months post-treatment, fixed orthodontic treatment provides broad, uniform OHRQoL improvement, particularly in functional domains, whereas orthognathic surgery yields superior psychosocial benefits, especially in social well-being and facial aesthetics, despite slower early functional recovery.

## RESUMEN

**Objetivo:**

La calidad de vida relacionada con la salud bucal (CVRSB) es un resultado clave en el tratamiento de las discrepancias dentofaciales. Este estudio de cohorte prospectivo tuvo como objetivo comparar las mejoras en la CVRSB tres meses después de completar el tratamiento de ortodoncia fija frente a la cirugía ortognática en pacientes femeninas iraníes.

**Materiales y métodos:**

Se incluyeron un total de 101 pacientes femeninas: 71 adolescentes (de 14 a 17 años) sometidas a tratamiento de ortodoncia fija y 30 mujeres jóvenes (de 19 a 35 años) sometidas a cirugía ortognática combinada por maloclusión esquelética. La CVRSB se evaluó al inicio del estudio y tres meses después del tratamiento mediante las versiones en persa del Cuestionario de Percepción Infantil (CPQ) para ambos grupos y el Cuestionario de Calidad de Vida Ortognática (OQLQ) para el grupo quirúrgico. Los cambios intra e intergrupales se analizaron mediante pruebas t pareadas e independientes (p < 0,05).

**Resultados:**

Ambos tratamientos mejoraron significativamente la calidad de vida relacionada con la salud bucal (CVRSB) en general. El grupo de ortodoncia mostró reducciones significativas en los cuatro dominios del CPQ y en la puntuación total (p < 0,001). El grupo quirúrgico presentó mejoras significativas en el bienestar emocional, el bienestar social y la puntuación total del CPQ (p < 0,001), pero no en los síntomas orales (p = 0,213) ni en las limitaciones funcionales (p = 0,284). Las puntuaciones del OQLQ en el grupo quirúrgico mejoraron en todos los dominios (p < 0,01).

**Conclusión:**

Pasados tres meses, el tratamiento de ortodoncia fija proporciona una mejora amplia y uniforme de la CVRSB, especialmente en los dominios funcionales, mientras que la cirugía ortognática ofrece beneficios psicosociales superiores, sobre todo en el bienestar social y la estética facial, a pesar de una recuperación funcional inicial más lenta.

## INTRODUCTION

Oral health-related quality of life (OHRQoL) is a crucial outcome in dental and maxillofacial treatment, reflecting not only clinical success but also the psychosocial and functional impact of interventions on patients’ daily lives ^1^^,^^2^. Malocclusions and dentofacial deformities impair OHRQoL through aesthetic concerns, functional limitations, emotional distress, and social interaction difficulties, especially in adolescents and young adults during formative periods of self-image and social development ^3^^,^^4^.

Orthodontic treatment and orthognathic surgery are common procedures for treating dentofacial discrepancies. Although both approaches aim to improve occlusion, facial appearance, and patient well-being, they differ considerably in indication, invasiveness, treatment burden, recovery process, and expected outcomes. Fixed orthodontic treatment is generally used to correct dental alignment problems and mild to moderate discrepancies, whereas orthognathic surgery combined with orthodontic treatment is indicated for more severe skeletal deformities that cannot be corrected by orthodontics alone ^5^^-^^7^. Therefore, the effects of these two treatment approaches on different domains of OHRQoL may not be identical.

Despite remarkable advantages, both treatment modalities have drawbacks that can temporarily impair patients' physical, emotional, and social well-being. Fixed orthodontic appliances can cause initial pain, ulceration, speech difficulty, and chewing problems ^8^^,^^9^. Orthognathic surgery is associated with more acute postoperative morbidity including swelling, neurosensory disturbance, dietary restrictions, and risk of complications, which may intensify emotional distress and social isolation for several weeks to months ^10^^,^^11^. These differences in treatment experience highlight the need to evaluate not only overall improvement, but also domain-specific changes in OHRQoL after each intervention.

Most previous studies have assessed OHRQoL after orthodontic treatment or orthognathic surgery separately. However, direct prospective comparisons between these two treatment pathways remain limited. This gap is clinically important because treatment planning often requires balancing functional needs, aesthetic expectations, psychosocial concerns, invasiveness, and recovery burden. In particular, some patients with borderline dentofacial discrepancies may be considered for either orthodontic camouflage or combined surgical-orthodontic correction ^12^^,^^13^. Therefore, comparing the OHRQoL effects of these modalities using standardized patient-reported outcome measures may provide more practical evidence for patient counseling, expectation management, and individualized treatment planning.

Furthermore, OHRQoL outcomes may be influenced by cultural and social factors. In Middle Eastern populations, attitudes toward facial aesthetics, gender roles, and social acceptability may shape patients’ expectations and perceptions of treatment outcomes ^14^^,^^15^. However, evidence from this region remains limited. Focusing on Iranian female patients may therefore provide useful insight into a population in which psychosocial and aesthetic concerns may play a particularly important role in treatment-related quality of life.

Accordingly, the present study used a prospective comparative approach to evaluate and compare changes in OHRQoL after fixed orthodontic treatment and orthognathic surgery among Iranian female patients. By assessing domain-specific changes before and three months after treatment, this study aimed to clarify whether these two treatment modalities produce different patterns of functional, emotional, and social improvement.

## MATERIALS AND METHODS

This prospective cohort study was conducted at the Dental Clinic of the School of Dentistry, Mashhad University of Medical Sciences, Iran. The study protocol was approved by the Ethics Committee of Mashhad University of Medical Sciences (code: IR.mums.sd.REC.1394.214). Written informed consent was obtained from all participants or their legal guardians.

### Participants

This study included 101 female patients divided into two groups:

- The orthodontic group (n = 71): patients aged 14-17 years with no significant maxillomandibular discrepancy, scheduled for fixed orthodontic treatment.
- The orthognathic surgery group (n = 30): patients aged 19-35 years with skeletal malocclusion, planned for combined orthodontic-orthognathic treatment, including pre- and postoperative orthodontic correction followed by orthognathic surgery.

Participants with chronic systemic diseases, medication use, previous orthodontic treatment, craniofacial anomalies such as cleft lip/palate, extensive dental caries, or periodontal diseases were excluded.

### Sample Size Calculation

Based on a formula for comparing means between two independent groups, with a type I error of 5% and type II error of 20%, a minimum of 34 participants per group was required. To account for potential attrition, 35 patients were initially targeted per group. Participants were selected using a purposive sampling method.

### Data collection 

Two validated questionnaires were used to assess OHRQoL of the patients. The Child Perception Questionnaire (CPQ), in its validated Persian version, consists of 48 items distributed across four domains: oral symptoms, functional limitations, emotional well-being, and social well-being. Responses are recorded on a 5-point Likert scale (0 = “never,” 1 = “once or twice,” 2 = “sometimes,” 3 = “often,” 4 = “every day/almost every day”). Domain scores are obtained by summing the respective item responses, with higher scores indicating greater negative impact and thus poorer OHRQoL. The validity and reliability of the Persian CPQ had previously been confirmed in a pilot study conducted in the Iranian population ^16^.

The Orthognathic Quality of Life Questionnaire (OQLQ) comprises 22 items categorized into four domains: facial aesthetics, oral function, awareness of facial deformity, and social aspects. Items are scored on a 5-point scale ranging from 1 (“bothers you a little”) to 4 (“bothers you a lot”), with an additional “not at all” option scored as 0. Total scores range from 0 to 88, where lower scores reflect better orthognathic-specific quality of life. The Persian translation of the OQLQ had its validity and reliability established in a prior thesis study ^17^.

After obtaining written informed consent from the patients or their legal guardian, baseline questionnaires were administered. Both groups completed the CPQ before treatment. The surgical group also completed the OQLQ before surgery.

Follow-up assessments were conducted three months after the completion of fixed orthodontic treatment and three months after orthognathic surgery. The same questionnaires were re-administered under researcher supervision to ensure consistent and timely completion.

### Statistical Analysis

Data were analyzed using SPSS version 20. Descriptive data were presented using descriptive statistics as frequency, mean, and standard deviation. Normality of distribution was assessed using the Shapiro-Wilk test. Within-group pre-post comparisons of CPQ scores were analyzed using paired t-tests. Between-group comparisons were conducted using independent t-tests. A p-value < 0.05 was considered statistically significant.

## RESULTS

The cohort included two groups: an orthodontic group (n = 71) with the mean age 15.5 ± 1.1 years old and a surgical group (n = 30) with the mean age 22.1 ± 3.4 years old. Normality of the data was confirmed using the Shapiro-Wilk test.

Findings of the paired t-test suggested that in the orthodontic group, significant reductions were observed in all four domains as well as in the total CPQ score (P < 0.001). In the orthognathic surgery group, the mean oral symptoms score and the functional limitations score decreased three months postoperatively; however, the differences were not statistically significant (p = 0.213 and p = 0.284, respectively). In contrast, emotional well-being, social well-being and total CPQ scores decreased significantly (P < 0.001) (Figure 1).

**Figure 1:**
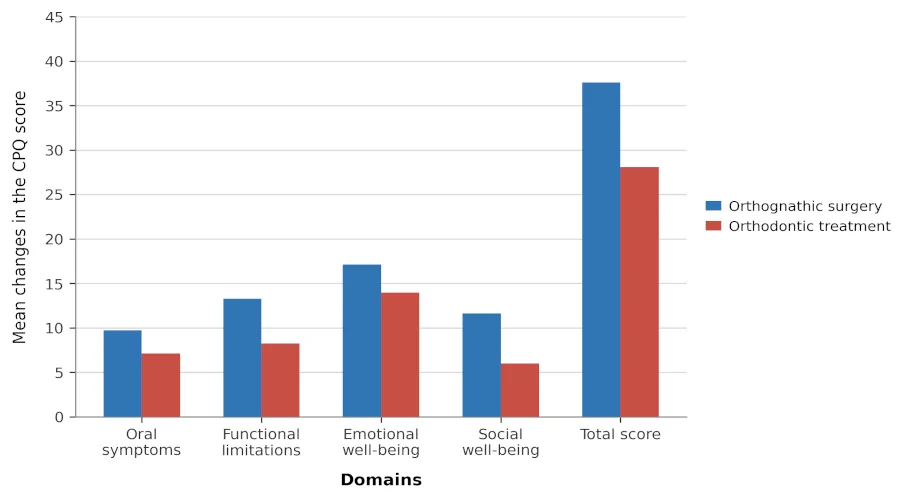
Comparison of the changes in CPQ scores among the study groups

The magnitude of improvement in the CPQ differed across domains when the two treatment groups were compared (Table 1). Patients treated with orthognathic surgery exhibited significantly greater improvement in the oral symptoms domain than those who underwent orthodontic treatment (P = 0.013). Improvement in functional limitations and emotional well-being was also larger in the orthognathic surgery group, although the difference did not reach statistical significance. In contrast, improvements in social well-being were significantly greater in the orthognathic surgery group (P = 0.004). Overall total CPQ score improvement was greater after surgery, but the difference was not statistically significant (P = 0.662).

**Table 1:** Comparison of mean changes in CPQ domain scores between the two study groups

Domain	Orthognathic surgery group Mean ± SD	Orthodontic group Mean ± SD	p-value*
Oral symptoms	9.75 ± 2.27	7.11 ± 6.65	0.013
Functional limitations	13.28 ± 2.67	8.25 ± 5.69	0.258
Emotional well-being	17.11 ± 16.43	13.98 ± 11.30	0.118
Social well-being	11.65 ± 8.93	6.02 ± 3.69	0.004
Total CPQ score	37.61 ± 30.30	28.09 ± 27.32	0.662

* Independent t-test

The findings of the OQLQ in the surgery group demonstrated statistically significant improvements across all domains three months postoperatively (Table 2). The greatest mean improvements were observed in social aspects (10.93 ± 10.94) and facial aesthetics (9.50 ± 5.95), followed by awareness of facial deformity (3.47 ± 4.97) and oral function (3.13 ± 6.95). All domains improved significantly, resulting in a significant reduction in the total OQLQ score (27.03 ± 24.44; p < 0.001), reflecting improved orthognathic-specific quality of life.

**Table 2:** The mean changes in the OQLQ scores in the orthognathic surgery group

Domain	Mean ± SD	p-value*
Facial aesthetics	9.50 ± 5.95	< 0.001
Oral function	3.13 ± 6.95	0.020
Awareness of facial deformity	3.47 ± 4.97	< 0.001
Social aspects	10.93 ± 10.94	< 0.001
Total OQLQ score	27.03 ± 24.44	< 0.001

* One-sample t-test

## DISCUSSION 

This current study compared the changes in OHRQoL following fixed orthodontic therapy and combined orthodontic-orthognathic surgery in a sample of Iranian female patients. Both orthodontic treatment and orthognathic surgery led to improvements in OHRQoL at the three-month follow-up. While fixed orthodontic treatment primarily benefited oral symptoms and functional limitations, orthognathic surgery caused enhancements in psychosocial domains including emotional and social well-being.

In the present study, orthodontic therapy produced reductions across all CPQ domains, with particularly highlighted effects on oral symptoms and functional limitations. This aligns with a recent prospective cohort study which reported that orthodontic treatment in patients under 18 years led to a substantial mean reduction in Malocclusion Impact Questionnaire (MIQ) scores ^18^. Similarly, a cross-sectional analysis of adolescents during the initial six months of fixed orthodontic therapy reported early enhancements in functional and emotional domains ^9^. Moreover, the efficacy of fixed orthodontics in OHRQoL improvement in the present study is corroborated by Jamilian et al. who documented significant OHRQoL and self-esteem improvements following non-surgical treatment ^19^. These findings reinforce that orthodontic interventions offer reliable, broad-spectrum relief for adolescents with dentoalveolar discrepancies.

On the other hand, orthognathic surgery, applied to older patients with severe skeletal deformities, demonstrated more selective CPQ improvements, with non-significant changes in oral symptoms and functional limitations, but remarkable improvements in emotional and social well-being. The significant postoperative improvement in facial aesthetics and social aspects is consistent with previous studies. Rustemeyer et al. identified the largest effect sizes in aesthetic and social domains following surgery, noting that these factors often outweigh functional gains in their impact on overall quality of life ^20^. Similarly, Al-Sebaei et al. reported the greatest postoperative changes in social aspects and aesthetic satisfaction, particularly among female patients ^21^.

In line with current findings, Antoun et al. found that orthognathic surgery patients showed the greatest improvement in quality of life compared to those receiving other treatments ^22^. Additionally, some studies noted that the orthodontic phase can temporarily reduce a patient’s well-being, while surgery itself tends to provide a quick boost in confidence and social comfort ^23^^,^^24^. This pattern supports the present results, where surgery led to stronger emotional and social benefits. Even though the combined surgical-orthodontic process is longer and more demanding, it appears to offer unique and powerful rewards in psychological and social well-being.

The present findings suggest that emotional and social well-being improve faster than physical function. In this regard, a before after study using the Oral Health Impact Profile (OHIP) reported an initial postoperative deterioration in OHRQoL. However, by six months, scores returned to pre surgery levels, and after two years, they showed long lasting improvements, especially in psychological and social domains ^25^. Additionally, a cohort study corroborated long-term (24-month) significant improvement in patients' quality of life and aesthetic outcome following orthognathic surgery, which provided psychosocial benefits that outweighed temporary early functional limitations ^26^.

The OQLQ findings in the present study revealed the largest score reductions in facial aesthetics and social aspects. This pattern highlights orthognathic surgery's efficacy not just as a functional correction, but as a powerful intervention against the psychosocial burden of facial deformity. Notably, the between-group analyses indicated superior surgical gains in oral symptoms and social well-being, suggesting that for borderline cases, the skeletal corrections afforded by surgery may yield disproportionate psychosocial dividends over orthodontic camouflage. In line with these findings, a systematic review and meta-analysis of Class III malocclusions favored orthognathic approaches for aesthetic and soft-tissue outcomes, indirectly supporting enhanced social OHRQoL ^27^.

A critical contribution of this study is its focus on an Iranian female population. Cultural attitudes towards beauty, gender roles, and social acceptance profoundly modulate patient expectations and the valuation of treatment outcomes. The exceptionally large improvement in social well-being within the current surgical group may reflect the heightened social pressures and importance placed on facial harmony within this cultural context. This aligns with findings by Al-Sebaei, who observed marked gender differences, with women showing greater psychosocial gains from orthognathic surgery than men ^21^. The present all-female cohort likely amplified this effect, suggesting that for young women in this region, surgical correction of a skeletal deformity may be particularly liberating from social constraints.

The current findings suggest that patients whose quality of life is predominantly impaired by social anxiety, poor self-image, and aesthetic concern will likely experience a more profound and targeted benefit from the orthognathic surgical pathway, despite its greater invasiveness and recovery burden. However, the exclusive inclusion of female patients, while revealing for gender-specific analysis, limits the generalizability of findings to males. Furthermore, the three-month follow-up captures early recovery but misses potential long-term trajectories, as evidenced by studies showing peak surgical benefits at 12-24 months ^26^. Future research should pursue longitudinal, multi-center studies with larger, gender-balanced cohorts to validate these patterns. Investigating the influence of specific malocclusion types and surgical techniques on domain-specific OHRQoL outcomes would provide further granularity for personalized care. 

From a clinical perspective, the findings of this study may help clinicians tailor treatment recommendations according to patients’ main concerns, expectations, and quality-of-life priorities. Patients whose primary complaints are related to oral symptoms, chewing difficulty, or functional limitations may experience meaningful improvement after fixed orthodontic treatment, particularly when skeletal discrepancies are mild to moderate. In contrast, patients with severe dentofacial deformities whose quality of life is mainly affected by facial appearance, self-confidence, emotional distress, or social interaction may benefit more from orthognathic surgery, despite its greater invasiveness and temporary postoperative morbidity.

These findings are also useful for pre-treatment counseling and expectation management. Clinicians should inform patients that orthognathic surgery may produce earlier and more pronounced psychosocial benefits, especially in facial aesthetics and social well-being, while functional recovery may be slower during the early postoperative period. Conversely, fixed orthodontic treatment may provide broader and more gradual improvements in daily oral function and general well-being. Therefore, treatment planning should not rely only on cephalometric measurements, occlusal findings, or clinician-based assessments, but should also incorporate patient-reported concerns and domain-specific OHRQoL outcomes. This patient-centered approach may improve shared decision-making, especially in borderline cases where both orthodontic camouflage and surgical-orthodontic correction may be considered.

## CONCLUSION

Within the limitations of this study, both fixed orthodontic treatment and orthognathic surgery are effective in improving OHRQoL of patients with dentofacial discrepancies. Orthodontic treatment offers robust, across-the-board improvements, effectively enhancing daily oral function and well-being. In contrast, orthognathic surgery delivers a more targeted and transformative benefit, achieving its pinnacle effect in the psychosocial domains, notably in social confidence and satisfaction with facial aesthetics. This differential efficacy profile provides essential evidence for tailoring treatment recommendations to the individual patient’s chief concerns and priorities.
